# Assessment of clinical outcomes in patients with fibromyalgia: Analysis from the UK Medical Cannabis Registry

**DOI:** 10.1002/brb3.3072

**Published:** 2023-05-18

**Authors:** Claire Wang, Simon Erridge, Carl Holvey, Ross Coomber, Azfer Usmani, Mohammed Sajad, Rahul Guru, Wendy Holden, James J. Rucker, Michael W. Platt, Mikael H. Sodergren

**Affiliations:** ^1^ Medical Cannabis Research Group, Department of Surgery and Cancer Imperial College London London UK; ^2^ Sapphire Medical Clinics London UK; ^3^ St. George's Hospital NHS Trust London UK; ^4^ Department of Psychological Medicine Kings College London London UK; ^5^ South London & Maudsley NHS Foundation Trust London UK

**Keywords:** cannabidiol, fibromyalgia, medical cannabis, tetrahydrocannabinol

## Abstract

**Introduction:**

: There are limited therapeutic options for individuals with fibromyalgia. The aim of this study is to analyze changes in health‐related quality of life and incidence of adverse events of those prescribed cannabis‐based medicinal products (CBMPs) for fibromyalgia.

**Methods:**

: Patients treated with CBMPs for a minimum of 1 month were identified from the UK Medical Cannabis Registry. Primary outcomes were changes in validated patient‐reported outcome measures (PROMs). A *p*‐value of <.050 was deemed statistically significant.

**Results:**

: In total, 306 patients with fibromyalgia were included for analysis. There were improvements in global health‐related quality of life at 1, 3, 6, and 12 months (*p* < .0001). The most frequent adverse events were fatigue (*n* = 75; 24.51%), dry mouth (*n* = 69; 22.55%), concentration impairment (*n* = 66; 21.57%), and lethargy (*n* = 65; 21.24%).

**Conclusion:**

: CBMP treatment was associated with improvements in fibromyalgia‐specific symptoms, in addition to sleep, anxiety, and health‐related quality of life. Those who reported prior cannabis use appeared to have a greater response. CBMPs were generally well‐tolerated. These results must be interpreted within the limitations of study design.

## Graphical abstract

A case series was analysed of 306 patients with fibromyalgia enrolled in the UK Medical Cannabis Registry. Cannabis‐based medicinal product treatment was associated with improvements in fibromyalgia‐specific symptoms, in addition to sleep, anxiety and health related quality of life. CBMPs were generally well‐ tolerated, however those with fibromyalgia may be more sensitive to their adverse effects compared to those with other conditions where they are prescribed.

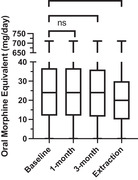

## 1 INTRODUCTION

Fibromyalgia is a syndrome defined as chronic widespread musculoskeletal pain across multiple points of the body in addition to cardinal signs and symptoms, such as sleep disturbance, confirmed with palpation of multiple tender points during a physical exam (Bair & Fibromyalgia, 2020; Bellato et al., 2012; Siracusa et al., 2021). Other key symptoms are cognitive dysfunction sometimes referred to as “fibro fog” (Kravitz & Katz, 2015), as well as digestive dysfunction (Clauw, 2015). In American and European populations, the mean prevalence is 4%, with a 2:1 female to male ratio (Ablin & Sarzi‐Puttini, 2020; Cameron & Hemingway, 2020). Due to the burden of pain and supplementary symptoms, patients are frequently limited in their ability to work and conduct activities of daily living (Arnold et al., 2008; Palstam & Mannerkorpi, 2017). Patients with fibromyalgia also report a high prevalence of psychiatric comorbidity, especially compared to other chronic pain syndromes (Arnold et al., 2008; Verbunt et al., 2008). Moreover, poor sleep quality contributes to fatigue, which affects 76% of fibromyalgia patients (Choy, 2015; Vincent et al., 2013).

Fibromyalgia is a central sensitivity syndrome, where patients with the condition have a lower pain threshold and increased perception of pain (Bellato et al., 2012; Siracusa et al., 2021; Yunus, 1992). This is the result of neuronal signal amplification in the central nervous system, characterized by excitability of spinal cord neurons where following a pain stimulus, successive stimuli of identical intensity are perceived more intensely (Li et al., 1999). This expression of neuroplasticity is a normal phenomenon, but in fibromyalgia this becomes a pathological process (Staud et al., 2001).

The treatment of fibromyalgia is focused on a biopsychosocial model. Nonpharmacological management is centered around psychological therapies, such as cognitive behavioral therapy and psychotherapy, or exercise‐based approaches (Berger et al., 2020). These treatments are supported by moderate strength evidence (Busch et al., 2007; Creamer et al., 2000). Multiple Cochrane systematic reviews on pharmacological approaches to fibromyalgia, however, have concluded that the current existing evidence is heterogenous and of poor quality, and currently does not support the use of any pharmacological treatments in fibromyalgia (Cooper et al., 2017; Thorpe et al., 2018; Walitt et al., 2016; Welsch et al., 2018). However, many patients are affected with refractory symptoms, despite best nonpharmacological treatment (Kwiatek, 2017). Tricyclic antidepressants such as amitriptyline have been observed to have a role in improving mental state, pain, and sleep (Macfarlane et al., 2017). Opioids are also commonly prescribed in fibromyalgia, but there is a paucity of evidence describing their positive effects (Carville et al., 2008; Goldenberg et al., 2016; Painter & Crofford, 2013). Furthermore, opioids carry a significant profile of adverse effects and risk of abuse (Benyamin et al., 2008). There is subsequently a need to develop novel therapeutic options that address the broad spectrum of symptoms that affect those with fibromyalgia.

There are increasing reports of self‐administered cannabis use for symptom relief in fibromyalgia (Boehnke et al., 2021; Wipfler et al., 2019), suggesting it to be an area worthy of investigation. Cannabis plants contain over 140 pharmacologically active cannabinoids, compounds that interact with the endocannabinoid system, a cell‐signaling system with an important role in the central nervous system and inflammatory response (Gonçalves et al., 2021). It has been hypothesized that a deficiency of endocannabinoid activity may play a role in the pathophysiology of fibromyalgia (Cameron & Hemingway, 2020; Tzadok & Ablin, 2020). The effect of cannabinoids is primarily mediated by type 1 and type 2 cannabinoid receptors (CB1 and CB2), through inhibiting synaptic transmission of gamma‐aminobutyric acid and glutamate (Barrie & Manolios, 2017). Activation of CB1 and CB2 receptors are considered to produce antinociceptive and anti‐inflammatory effects, respectively, in chronic pain (Anthony et al., 2020). CB1 receptors are highly expressed in the central nervous system, with high density in areas such as the prefrontal cortex and the amygdala, which is associated with central nociceptive processing (Vučković et al., 2018). CB2 receptors are predominantly found in peripheral tissues and immune cells where they play a role modulating cytokine release and immune cell migration (Smith & Wagner, 2014).

The two most abundant cannabinoids found in the cannabis plant are delta‐9‐tetrahydrocannabinol (THC) and cannabidiol (CBD) (Berger et al., 2020). CBD inhibits fatty acid binding ligands that transport endocannabinoid anandamide intracellularly, therefore inhibiting its hydrolysis by fatty acid amide hydrolase (Bisogno et al., 2001). Anandamide is a CB1 agonist, producing an antinociceptive effect (Bisogno et al., 2001; Pertwee & Ross, 2002; Romero et al., 2013). CBD is also an agonist of selective serotonin 1A (5HT1A) receptors, which produces anxiolytic effects by action in the midbrain (Campos & Guimarães, 2008). CBD has also demonstrated analgesic and anti‐inflammatory properties through interaction with transient vanilloid subtype 1 receptors (TRPV1), nonselective cation channels that detect noxious stimuli (Costa et al., 2004; Fischer et al., 2020). CBD binds directly to TRPV1 receptors and evokes a refractory state of desensitization to noxious stimuli (Costa et al., 2004). Anandamide is also a TRPV1 agonist with high binding affinity but a reduced activating effect (Lizanecz et al., 2006; Ross et al., 2001). Thus, as CBD inhibits anandamide hydrolysis, this potentiates the effects of TRPV1 desensitization. THC is a CB1 receptor partial agonist, and results in reduced neurotransmission of nociceptive signals (Cravatt & Lichtman, 2004). THC also shows psychoactive effects, such as emotional regulation, suspected to be the result of downregulation of neurotransmission in the basal ganglia, cerebellum, and hippocampus (Akirav, 2011). This may enhance descending pain pathways reducing severity and impact (Urits et al., 2019).

With respect to clinical evidence, there is a paucity of high‐quality evidence of the effects of cannabis‐based medicinal products (CBMPs) (Cameron & Hemingway, 2020). However, studies have demonstrated that CBMPs could contribute to improvements to pain, sleep, and mood in patients with chronic pain (Wang et al., 2021), and in fibromyalgia specifically (Mayorga‐Anaya et al., 2021; Mazza, 2021; van de Donk et al., 2019). Moreover, a recent systematic review of the use of cannabinoid compounds in fibromyalgia also found that there was a minimal associated adverse event profile (Khurshid et al., 2021). However, studies of fibromyalgia have focused on pain‐specific outcomes only, with limited data on the effects on additional sensitization symptoms, such as anxiety. Moreover, they are affected by small sample sizes and significant heterogeneity. The aim of this study is therefore to assess the outcomes of patients prescribed CBMPs in the setting of fibromyalgia on fibromyalgia‐specific symptoms, health‐related quality of life, anxiety, and sleep. Secondary aims include changes in opioid consumption during treatment with CBMPs, as well as evaluation of adverse events.

## 2 METHODS

### 2.1 Study design

This is an uncontrolled case series including patients treated with CBMPs for a minimum of 1 month for fibromyalgia who were identified from the UK Medical Cannabis Registry (UKMCR). All participants provided their informed consent when enrolling in the registry and are enrolled consecutively. Formal ethical approval was not required for this registry study. STROBE guidelines were followed for study reporting (von Elm et al., 2007).

### 2.2 Participants and setting

The UKMCR was established in 2019, collecting pseudonymized data from patients prescribed CBMPs in the United Kingdom and Channel Islands at Sapphire Medical Clinics, a private healthcare setting (Erridge et al., 2021). Over 92% of patients at the clinic are enrolled in the UKMCR. This study includes patients who have fibromyalgia as their primary indication for treatment with CBMPs. Patients who had not completed a baseline assessment or had not received a prescription for CBMPs longer than 1 month were excluded from the study.

### 2.3 Data selection

Baseline data including patient demographics and comorbidities were collected by clinicians. The Charlson comorbidity index, a commonly used assessment to determine baseline comorbidity in population studies, was subsequently calculated (Quan et al., 2011). Drug, alcohol, and cannabis history was also collected and used to ascertain tobacco and cannabis use status. Lifetime smoking exposure was reported in pack years, while weekly alcohol intake was recorded in units. Current quantity of cannabis and lifetime cannabis consumed were recorded in grams per day and gram years, respectively. Cannabis gram years is calculated as follows: mean cannabis consumption (g/day) × number of years used (Wetherill et al., 2016).

Concurrent medications were electronically recorded with start and end dates of prescriptions. These were mapped to SNOMED‐CT terminology to ensure correct recording throughout. CBMP prescriptions were also recorded during treatment, including the manufacturer, route of administration, formulation, THC and CBD concentration, and dose (mg) per 24 h. The maximally titrated formulation and concentration of THC and CBD were made available for the present analysis.

Patient‐reported outcome measures (PROMs) assessing health‐related quality of life were collected electronically at baseline, 1 month, 3 months, 6 months, and 12 months.

### 2.4 Patient‐reported outcome measures

#### 2.4.1 Fibromyalgia Symptom Severity

Fibromyalgia Symptom Severity is a diagnostic criterion specific to fibromyalgia that combines widespread pain index (WPI) with symptom severity scale (SSS) scores (Fibromyalgia Network, n.d.; Wolfe et al., 2011). Patients indicate the areas they have felt pain in the past week and the total number of areas affected, producing a WPI score (Fibromyalgia Network, n.d.). Then, in a two‐part evaluation, patients indicate symptom severity in the past week to give an SSS score (range: 0–12) (Fibromyalgia Network, n.d.). Together these scores are combined to give a total Fibromyalgia Symptom Severity between 0 and 31. Higher scores are associated with increased severity of both pain and auxiliary symptoms (Wolfe et al., 2010).

#### 2.4.2 Single‐Item Sleep Quality Scale

Single‐Item Sleep Quality Scale (SQS) is a single‐item scale that rates the patient's quality of sleep in the previous 7 days from 0 (*terrible*) to 10 (*excellent*) (Snyder et al., 2018).

#### 2.4.3 Patients’ Global Impression of Change

Patients’ Global Impression of Change (PGIC) is a numerical rating scale that reflects the patient's perception of improvement in their condition compared to baseline, and belief about the efficacy of treatment (Norton et al., 2009). The patient rates their change according to statements from 1 (*no change or worse*) to 7 (*a great deal better, considerable improvement*) (Norton et al., 2009).

#### 2.4.4 General Anxiety Disorder Scale

General Anxiety Disorder Scale (GAD‐7) is a seven‐item scale of the severity of generalized anxiety disorder symptoms, where the participant responds on a numerical rating Likert scale from 0 to 3 corresponding to the frequency of the symptoms (Löwe et al., 2008; Spitzer et al., 2006). The total score ranges from 0 to 21, categorized into mild (≥5), moderate (≥10), and severe (≥15) anxiety (Löwe et al., 2008; Spitzer et al., 2006).

#### 2.4.5 Visual Analogue Scale‐Pain

The Visual Analogue Scale‐Pain (VAS‐Pain) asks patients to assess the severity of pain they experience at that moment on a 10‐cm visual scale from 0 (*no pain*) to 10 (*the most painful possible*) (Delgado et al., 2018). The minimum clinically significant difference in pain is approximately a 13‐mm change in the linear score (Sadovsky, 2002).

#### 2.4.6 EQ‐5D‐5L

EQ‐5D‐5L is a self‐reported questionnaire that evaluates health‐related quality of life (https://euroqol.org/eq‐5d‐instruments/eq‐5d‐5l‐about]). Patients score their quality of life from 1 (*no problems*) to 5 (*extreme problems*) on the day of completion in five areas: “Anxiety and Depression,” “Mobility,” “Self‐Care,” “Pain and Discomfort,” and “Usual Activities” (British National Formulary—NICE). An index score is calculated where optimum health is signified by 1, and a negative value represents an instance where the health‐related quality of life of the individual is deemed to be worse than death (van Hout et al., 2012). This index value is specific to a U.K. population and is the preferred assessment for health‐related quality of life by the National Institute for Health and Care Excellence (2019).

### 2.5 Opioid consumption analysis

Using the medication history of patients with opioid prescriptions and conversion factors detailed by the British National Formulary, a daily oral morphine equivalent was calculated in milligram per 24 h (British National Formulary—NICE).

### 2.6 Adverse events

Participants either completed adverse events electronically at the time of their event or were asked to record these either prior to completing PROMs or during routine clinician follow‐ups if otherwise unrecorded. The adverse events were classified according to the Common Terminology Criteria for Adverse Events version 4.0 (National Cancer Institute, 2009).

### 2.7 Statistical methods

PROMs data were analyzed at 1, 3, 6, and 12 months compared to baseline. Subgroup analysis according to previous cannabis status was only performed up until 6 months due to limited number of participants available at 12 months. Changes in consumption of prescribed opiates per 24 h were analyzed at 1 month, 3 ‐months, and end of follow‐up. A Shapiro–Wilk test was used to determine normality. Nonparametric data were presented as median (interquartile range [IQR]), and parametric data were presented as mean ± standard deviation (SD).

Paired statistical analysis was performed using a paired *t*‐test or Wilcoxon rank‐sum test, dependent upon whether the data were parametric or nonparametric, respectively. A *p*‐value of <.050 was deemed statistically significant. A Bonferroni correction was applied to *p*‐values to reduce the likelihood of Type I error. Effect size (*r*) was calculated for the Wilcoxon rank‐sum test by dividing the *Z*‐value by the square root of the number of participants. IBM Statistical Package for Social Sciences (version: 27.0.0.0 SPSS Inc., Armonk, NY, USA) was used for data analysis, and GraphPad Prism (version 9.0.0; San Diego, CA, USA) was used for visualization.

## 3 RESULTS

On February 15, 2022, 3546 patients were registered on the UKMCR, of which 3240 (91.37%) were excluded (baseline PROM not completed: *n* = 443, 12.49%; enrolled for <1 month: *n* = 270, 7.61%; and fibromyalgia was not the primary indication for therapy: *n* = 2527, 71.26%). After applying inclusion criteria, 306 patients with fibromyalgia were included in the present analysis. Of these, 253 (83.59%), 177 (58.42%), and 68 (22.22%) participants had been enrolled for a minimum of 3, 6, and 12 months, respectively. The baseline demographics for the cohort are detailed in full in Table 1.

**TABLE 1:** Baseline demographics of study participants (*n* = 306).

Patient demographics	*n* (%) or mean (± *SD*)
Gender	
Female	215 (70.26%)
Male	90 (29.41%)
Other	1 (0.33%)
Age	44.74 (± 12.29)
Body mass index (kg/m^2^)	29.29 (± 8.10)
Employment	
Unemployed	163 (53.27%)
Not recorded	58 (18.95%)
Professional	25 (8.17%)
Managers	12 (3.92%)
Clerical support workers	10 (3.27%)
Technicians and associate professionals	10 (3.27%)
Service and sales workers	9 (2.94%)
Elementary occupations	8 (2.61%)
Craft and related trades workers	6 (1.96%)
Plant and machine operators and assemblers	3 (0.98%)
Skilled agricultural, forestry, and fishery workers	2 (0.65%)

The median Charlson comorbidity index value for the cohort was 1.00 (0.00–6.00). Additional recorded comorbidities included anxiety/depression (*n* = 166; 54.25%), arthritis (*n* = 98; 32.0%), hypertension (*n* = 38;12.42%), epilepsy (*n* = 5; 1.63%), and endocrine dysfunction (*n* = 40; 13.07%).

At baseline, 116 (37.91%) patients were ex‐smokers; 98 (32.03%) patients had never smoked; 91 (29.74%) were current smokers; and one (0.33%) patient had no data available. Of 299 patients where data were available, median weekly alcohol consumption was 0.00 units (range: 0.00–25.00). At the point of enrolment, just under half of all patients (*n* = 149; 48.69%) were current users of cannabis; 116 (37.91%) were cannabis naïve; and 41 (13.40%) were ex‐users of cannabis. The median lifetime cannabis consumption was 5.00 (1.00–12.50) gram years.

### 3.1 Cannabis‐based medicinal products

The maximally titrated CBMP data were available for 290 (94.77%) patients. Oral/sublingual oil preparations were prescribed to 118 (40.69%) patients, while 35 (12.07%) were prescribed dry plant (vaporized) preparations only, and 137 (47.24%) had a combination of both. The median dose of THC was 100.00 (20.00–195.00) mg/day and the median dose of CBD was 20.00 (20.00–35.00) mg/day. The most common CBMP oils were Adven^®^ 20 THC and Adven^®^ 50 CBD (Curaleaf International, Guernsey, UK). The most common dried flower preparation was Adven^®^ EMT1 19% THC (Curaleaf International).

### 3.2 Patient‐reported outcome measures

The results of the PROMs analysis are detailed in full in Table 2. There was improvement in the EQ‐5D‐5L index value at all stages of follow up (1, 3, 6, and 12 months) compared to baseline (*p* < .001). There were improvements compared to baseline at 1‐, 3‐, and 6‐month follow‐ups in Fibromyalgia Symptom Severity, SQS, EQ‐5D‐5L Self‐Care, EQ‐5D‐5L Pain and Discomfort, EQ‐5D‐5L Anxiety and Depression, and EQ‐5D‐5L Usual Activities domains, in addition to the VAS‐Pain (*p* < .050). There was an increase in PGIC scores from 3‐ to 6‐month follow‐up from 5.00 (4.00–6.00) to 6.00 (5.00–6.00).

**TABLE 2:** Paired baseline and follow‐up scores for outcome measures: Fibromyalgia Symptom Severity, Single‐Item Sleep Quality Scale (SQS), Patients’ Global Impression of Change (PGIC), General Anxiety Disorder‐7 (GAD‐7), Visual Analogue Scale‐Pain (VAS‐Pain), and EQ‐5D‐5L index value and subcomponents at 1, 3, 6, and 12 months.

PROMs	Follow‐up	*n*	Scores at baseline	Scores at follow‐up	*p*‐value	Effect size (*r*)
Fibromyalgia Symptom Severity	1 month	231	23.00 (19.00–28.00)	20.00 (15.00–25.00)	**<.001**	–.49
	3 months	170	22.00 (18.00–27.00)	19.00 (15.00–24.00)	**<.001**	–.21
	6 months	100	20.00 (16.25–25.00)	17.00 (14.00–24.00)	**<.001**	–.39
	12 months	27	20.00 (16.00–27.00)	17.00 (13.00–21.00)	.999	–.43
SQS	1 month	247	3.00 (2.00–5.00)	5.00 (3.00–7.00)	**<.001**	–.29
	3 months	189	4.00 (2.00–6.00)	6.00 (3.00–7.00)	**<.001**	–.16
	6 months	111	4.00 (3.00–6.00)	6.00 (4.00–8.00)	**<.001**	–.18
	12 months	34	4.50 (3.00–6.00)	6.00 (4.00–8.00)	.360	–.09
PGIC	1 month	245	–	5.00 (4.00–6.00)	–	–
	3 months	183	–	5.00 (4.00–6.00)	–	–
	6 months	116	–	6.00 (5.00–6.00)	–	–
	12 months	37	–	6.00 (5.00–6.00)	–	–
GAD‐7	1 month	252	8.00 (3.00–14.00)	6.00 (3.00–10.00)	**<.001**	–.30
	3 months	188	7.00 (3.00–12.00)	6.00 (2.25–9.00)	**<.001**	–.27
	6 months	114	6.00 (2.00–11.00)	5.00 (1.00–9.00)	.320	–.25
	12 months	37	4.00 (2.00–10.50)	3.00 (1.00–6.00)	.999	–.34
VAS‐Pain	1 month	246	7.00 (6.00–8.00)	7.00 (5.00–8.00)	**<.001**	–.35
	3 months	183	7.00 (6.00–8.00)	6.00 (5.00–8.00)	**<.001**	–.37
	6 months	110	7.00 (5.00–8.00)	6.00 (4.00–7.00)	**<.001**	–.32
	12 months	34	7.00 (5.75–8.00)	7.00 (4.00–8.00)	.999	–.18
EQ‐5D‐5L Mobility	1 month	250	3.00 (2.00–4.00)	3.00 (2.00–3.00)	**<.001**	–.27
	3 months	186	3.00 (2.00–4.00)	3.00 (2.00–3.00)	.200	–.20
	6 months	113	3.00 (2.00–4.00)	2.00 (2.00–3.00)	**<.001**	–.33
	12 months	36	3.00 (2.00–4.00)	3.00 (2.00–3.00)	.999	–.26
EQ‐5D‐5L Self‐Care	1 month	250	3.00 (2.00–3.00)	2.00 (1.00–3.00)	**<.001**	–.27
	3 months	186	2.00 (2.00–3.00)	2.00 (1.00–3.00)	**<.001**	–.24
	6 months	113	2.00 (1.50–3.00)	2.00 (1.00–3.00)	**<.001**	–.33
	12 months	36	2.00 (2.00–3.00)	2.00 (1.00–3.00)	.160	–.48
EQ‐5D‐5L Usual Activities	1 month	250	3.00 (3.00–4.00)	3.00 (2.00–3.00)	**<.001**	–.38
	3 months	186	3.00 (2.00–4.00)	3.00 (2.00–3.00)	**<.001**	–.34
	6 months	113	3.00 (2.00–4.00)	3.00 (2.00–3.00)	**<.001**	–.35
	12 months	36	3.00 (2.25–4.00)	3.00 (2.00–3.00)	.999	–.31
EQ‐5D‐5L Pain and Discomfort	1 month	250	4.00 (3.00–4.00)	3.00 (3.00–4.00)	**<.001**	–.49
	3 months	186	3.00 (3.00–4.00)	3.00 (2.75–4.00)	**<.001**	–.44
	6 months	113	4.00 (3.00–4.00)	3.00 (2.00–3.00)	**<.001**	–.53
	12 months	36	4.00 (3.00–4.00)	3.00 (3.00–3.00)	.080	–.50
EQ‐5D‐5L Anxiety and Depression	1 month	250	3.00 (2.00–4.00)	2.00 (2.00–3.00)	**<.001**	–.27
	3 months	186	3.00 (2.00–3.00)	2.00 (1.00–3.00)	**<.001**	–.25
	6 months	113	2.00 (1.00–3.00)	2.00 (1.00–3.00)	**<.001**	–.40
	12 months	36	2.00 (1.00–3.00)	2.00 (1.00–3.00)	.999	–.32
EQ‐5D‐5L Index Value	1 month	250	0.33 (0.08–0.54)	0.53 (0.27–0.63)	**<.001**	–.39
	3 months	186	0.34 (0.10–0.56)	0.54 (0.26–0.65)	**<.001**	–.34
	6 months	113	0.38 (0.18–0.62)	0.56 (0.41–0.69)	**<.001**	–.39
	12 months	36	0.36 (0.21–0.61)	0.55 ( 0.40–0.67)	**<.001**	–.40

*Note*: *p* <.05.

#### 3.2.1 Previous cannabis users

Subgroup analysis in patients with history of cannabis consumption prior to treatment detailed in Table 3 showed statistically significant improvement in Fibromyalgia Symptom Severity, SQS, VAS‐Pain, and EQ‐5D‐5L index value at all follow‐up periods (1, 3, and 6 months) compared to baseline (*p* < .050). There was an increase in PGIC scores from 3‐ to 6‐month follow‐up from 5.00 (5.00–6.00) to 6.00 (5.00–6.00).

**TABLE 3:** Paired baseline and follow‐up scores for outcome measures: Fibromyalgia Symptom Severity, Single‐Item Sleep Quality Scale (SQS), Patients’ Global Impression of Change (PGIC), General Anxiety Disorder‐7 (GAD‐7), Visual Analogue Scale‐Pain (VAS‐Pain), and EQ‐5D‐5L index value and subcomponents at 1, 3, and 6 months in patients with previous cannabis use.

PROMs	Follow‐up	*n*	Scores at baseline	Scores at follow‐up	*p*‐value	Effect size (*r*)
Fibromyalgia Symptom Severity	1 month	156	24.00 (19.00–28.00)	20.50 (15.00–25.00)	**<.001**	–.54
	3 months	115	23.00 (19.00–28.00)	20.00 (15.00–24.00)	**<.001**	–.49
	6 months	68	21.00 (18.00–27.00)	17.00 (14.00–24.00)	**<.001**	–.52
	12 months	14	19.50 (15.00–27.25)	19.00 (13.00–23.25)	.999	–.22
SQS	1 month	167	3.00 (2.00–5.00)	5.00 (3.00–7.00)	**<.001**	–.16
	3 months	124	3.50 (2.00–6.00)	5.00 (3.00–7.00)	**<.001**	–.20
	6 months	75	4.00 (2.00–6.00)	6.00 (4.00–8.00)	**<.001**	–.11
	12 months	18	4.00 (3.00–6.00)	5.50 (3.75–8.00)	.999	–.06
PGIC	1 month	21	–	5.00 (5.00–5.00)	–	–
	3 months	163	–	5.00 (5.00–6.00)	–	–
	6 months	122	–	6.00 (5.00–6.00)	–	–
	12 months	78	–	6.00 (5.00–6.00)	–	–
GAD‐7	1 month	169	9.00 (4.00–15.00)	6.00 (3.00–10.00)	**<.001**	–.41
	3 months	126	9.00 (3.00–13.00)	6.00 (3.00–10.00)	**<.001**	–.35
	6 months	77	7.00 (3.00–12.00)	5.00 (2.00–9.00)	.440	–.29
	12 months	20	5.00 (2.00–11.75)	3.00 (1.00–6.00)	.999	–.15
VAS‐Pain	1 month	166	7.00 (6.00–8.00)	6.00 (5.00–8.00)	**<.001**	–.40
	3 months	124	7.00 (5.25–8.00)	6.00 (4.00–8.00)	**<.001**	–.37
	6 months	75	7.00 (5.00–8.00)	5.00 (4.00–7.00)	**<.001**	–.31
	12 months	18	7.00 (4.75–7.25)	6.50 (4.00–8.00)	.999	–.21
EQ‐5D‐5L Mobility	1 month	168	3.00 (2.00–4.00)	3.00 (2.00–3.00)	**<.001**	–.31
	3 months	125	3.00 (2.00–4.00)	3.00 (2.00–3.00)	.400	–.23
	6 months	76	3.00 (2.00–4.00)	2.00 (2.00–3.00)	**<.001**	–.43
	12 months	19	3.00 (2.00–4.00)	3.00 (2.00–3.00)	.999	–.25
EQ‐5D‐5L Self‐Care	1 month	168	3.00 (2.00–3.00)	2.00 (2.00–3.00)	**<.001**	–.30
	3 months	125	3.00 (2.00–3.00)	2.00 (1.00–3.00)	.280	–.24
	6 months	76	2.00 (2.00–3.00)	2.00 (1.00–3.00)	**<.001**	–.42
	12 months	19	2.00 (2.00–3.00)	2.00 (1.00–3.00)	.999	–.47
EQ‐5D‐5L Usual Activities	1 month	168	3.00 (3.00–4.00)	3.00 (2.00–4.00)	**<.001**	–.47
	3 months	125	3.00 (2.50–4.00)	3.00 (2.00–3.00)	**<.001**	–.34
	6 months	76	3.00 (2.00–4.00)	2.00 (2.00–3.00)	**<.001**	–.48
	12 months	19	3.00 (2.00–4.00)	3.00 (2.00–3.00)	.999	.22
EQ‐5D‐5L Pain and Discomfort	1 month	168	4.00 (3.00–4.00)	3.00 (3.00–4.00)	**<.001**	–.54
	3 months	125	4.00 (3.00–4.00)	3.00 (2.00–4.00)	**<.001**	–.43
	6 months	76	3.50 (3.00–4.00)	3.00 (2.00–3.00)	**<.001**	–.57
	12 months	19	3.00 (3.00–4.00)	3.00 (2.00–4.00)	.999	–.35
EQ‐5D‐5L Anxiety and Depression	1 month	168	3.00 (2.00–4.00)	2.00 (2.00–3.00)	**<.001**	–.33
	3 months	125	3.00 (2.00–4.00)	2.00 (1.00–3.00)	.120	–.27
	6 months	76	2.50 (1.25–3.75)	2.00 (1.00–3.00)	**<.001**	–.44
	12 months	19	3.00 (1.00−3.00)	2.00 (1.00–3.00)	.999	–.39
EQ‐5D‐5L Index Value	1 month	168	0.30 (0.04–0.54)	0.53 (0.26–0.63)	**<.001**	–.43
	3 months	125	0.32 (0.04–0.57)	0.53 (0.24–0.65)	**<.001**	–.36
	6 months	76	0.37 (0.11–0.62)	0.57 (0.44–0.71)	**<.001**	–.52
	12 months	19	0.45 (0.12–0.65)	0.55 (0.32–0.68)	.999	–.31

*Note*: *p* <.05.

#### 3.2.2 Cannabis naïve

PROMs analysis in patients who had no history of cannabis use prior to treatment detailed in Table 4 details improvements compared to baseline in EQ‐5D‐5L index values at 1 and 3 months (*p* < .050). There were improvements compared to baseline at 1 month in Fibromyalgia Symptom Severity scores (*p* < .001).

**TABLE 4:** Paired baseline and follow‐up scores for outcome measures: Fibromyalgia Symptom Severity, Single‐Item Sleep Quality Scale (SQS), Patients’ Global Impression of Change (PGIC), General Anxiety Disorder‐7 (GAD‐7), Visual Analogue Scale‐Pain (VAS‐Pain), and EQ‐5D‐5L index value and subcomponents at 1, 3, and 6 months in cannabis‐naïve patients.

PROMs	Follow‐up	*n*	Scores at baseline	Scores at follow‐up	*p*‐value	Effect size (*r*)
Fibromyalgia Symptom Severity	1 month	75	22.00 (17.00–27.00)	20.00 (15.00–25.00)	**<.001**	–.41
	3 months	55	21.00 (16.00–25.00)	18.00 (14.00–24.00)	.320	–.31
	6 months	32	20.00 (13.25–23.00)	18.00 (15.00–24.00)	.999	–.09
SQS	1 month	80	4.00 (2.00–5.75)	5.00 (3.00–7.00)	.080	–.13
	3 months	59	4.00 (3.00–6.00)	6.00 (3.00–8.00)	.560	–.14
	6 months	36	5.00 (3.00–7.00)	6.00 (4.00–8.00)	.999	–.03
PGIC	1 month	20	–	5.00 (3.50–5.00)	–	–
	3 months	82	–	5.00 (3.00–6.00)	–	–
	6 months	61	–	5.00 (4.00–6.00)	–	–
GAD‐7	1 month	83	6.00 (2.00–12.00)	6.00 (3.00–10.00)	.999	–.12
	3 months	62	5.00 (1.75–10.25)	4.50 (2.00–8.00)	.999	–.07
	6 months	37	4.00 (1.00–7.50)	4.00 (0.50–7.00)	.999	–.02
VAS‐Pain	1 month	80	7.00 (6.00–8.00)	7.00 (5.00–8.00)	.320	–.20
	3 months	59	7.00 (6.00–8.00)	6.00 (5.00–7.00)	**.040**	–.16
	6 months	35	6.00 (5.00–7.00)	6.00 (5.00–8.00)	.999	–.09
EQ‐5D‐5L Mobility	1 month	82	3.00 (2.00–4.00)	3.00 (2.00–3.00)	.999	–.18
	3 months	61	3.00 (2.00–4.00)	3.00 (2.00–3.00)	.999	–.14
	6 months	37	3.00 (2.00–4.00)	3.00 (2.00–3.00)	.999	–.14
EQ‐5D‐5L Self‐Care	1 month	82	3.00 (2.00–4.00)	3.00 (2.00–3.00)	.999	–.22
	3 months	61	2.00 (1.00–3.00)	2.00 (1.00–3.00)	.999	–.25
	6 months	37	2.00 (1.00–3.00)	2.00 (1.00–3.00)	.999	–.08
EQ‐5D‐5L Usual Activities	1 month	82	2.00 (1.00–3.00)	3.00 (2.00–3.00)	.999	–.21
	3 months	61	3.00 (2.00–4.00)	3.00 (2.00–3.00)	.320	–.33
	6 months	37	3.00 (2.00–4.00)	3.00 (2.00–4.00)	.578	–.09
EQ‐5D‐5L Pain and Discomfort	1 month	82	4.00 (3.00–4.00)	3.00 (3.00–4.00)	.080	–.35
	3 months	61	4.00 (3.00–4.00)	3.00 (3.00–4.00)	**<.001**	–.50
	6 months	37	4.00 (3.00–4.00)	3.00 (3.00–3.00)	.200	–.46
EQ‐5D‐5L Anxiety and Depression	1 month	82	3.00 (2.00–3.00)	2.50 (1.75–3.00)	.999	–.11
	3 months	61	2.00 (1.50–3.00)	2.00 (1.00–3.00)	.999	–.22
	6 months	37	2.00 (1.00–3.00)	2.00 (1.00–3.00)	.999	–.31
EQ‐5D‐5L Index Value	1 month	82	0.37 (0.20–0.54)	0.52 (0.27–0.63)	**<.001**	–.30
	3 months	61	0.38 (0.20–0.56)	0.56 (0.32–0.65)	**<.001**	–.31
	6 months	37	0.39 (0.22–0.62)	0.56 (0.36–0.64)	.320	–.12

*Note*: *p* <.05.

### 3.3 Oral morphine equivalent

**FIGURE 1:**
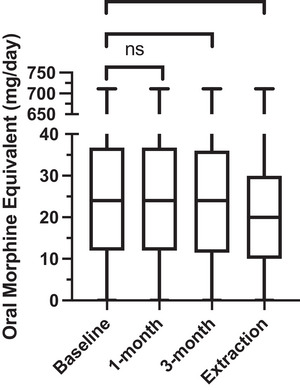
Baseline and follow‐up doses of oral morphine equivalent at 1 month, 3 months, and date of extraction with paired significance analysis (*p < .050; **p < .010; ***p < .001; *n* = 134).

At baseline, the median oral morphine equivalent dose of those prescribed opioid prescriptions at any point during the study (*n* = 134) was 24.00 (12.00–36.75) mg/day (Figure 1). At 1‐month follow‐up, median oral morphine equivalent dose was unchanged at 24.00 (12.00–36.75) mg/day (*p* = .180). The median oral morphine equivalent dose at 3‐month follow‐up was 24.00 (11.50–36.00) mg/day (*p* = .043), and at the end of follow‐up it was 20.00 (10.00–30.00) mg/day (*p* = .001). There was no increase in oral morphine equivalent dose for any patient after beginning CBMP treatment and no patients (*n* = 0; 0.0%) were newly commenced on opioid therapy during the study period.

### 3.4 Adverse events

Seventy‐two (23.53%) participants reported 979 adverse events, shown in Table 5. This is higher than the incidence (182.70%) of adverse events or proportion of patients (16.73%) affected by adverse events across all indications for enrolment in the UKMCR. The most common adverse events were fatigue (*n* = 75, 24.51%), dry mouth (*n* = 69, 22.55%), concentration impairment (*n* = 66, 21.57%), lethargy (*n* = 65, 21.24%), headache (*n* = 64, 20.92%), and somnolence (*n* = 59, 19.28%). Most adverse events were moderate (*n* = 436, 142.48%) or mild (*n* = 401, 131.05%). There were no (*n* = 0, 0.00%) life‐threatening/disabling adverse events. Regarding cannabis status, 41 (35.34%) of the cannabis naïve, 21 (13.09%) of the current user, and 10 (24.39%) of the ex‐user groups reported adverse events. Regarding administration, 38 (32.20%) of the oral/sublingual preparation only, seven (20.00%) of the dry plant (vaporized) only, and 27 (5.11%) of the combination (“both”) preparations groups reported adverse events.

**TABLE 5:** Reported adverse events.

Adverse events	Mild	Moderate	Severe	Life threatening/disabling	Total incidence (%)
Abdominal pain (upper)	15	9	6	0	30 (9.80%)
Amnesia	9	7	0	0	16 (5.23%)
Anorexia	12	7	3	0	22 (7.19%)
Anxiety	1	2	0	0	3 (0.98%)
Ataxia	14	10	1	0	25 (8.17%)
Blurred vision	13	6	1	0	20 (6.54%)
Bone pain	2	0	0	0	2 (0.65%)
Cognitive disturbance	18	21	1	0	40 (13.07%)
Concentration impairment	34	27	5	0	66 (21.57%)
Confusion	15	7	2	0	24 (7.84%)
Constipation	21	17	7	0	45 (14.71%)
Decreased weight	9	4	0	0	13 (4.25%)
Delirium	9	3	1	0	13 (4.25%)
Diarrhea	0	5	0	0	5 (1.63%)
Dizziness	15	25	9	0	49 (16.01%)
Dry mouth	59	10	0	0	69 (22.55%)
Dysgeusia	6	5	0	0	11 (3.59%)
Dyspepsia	19	11	1	0	31 (10.13%)
Dyspnea	0	1	0	0	1 (0.33%)
Euphoria	0	1	0	0	1 (0.33%)
Fall	4	1	0	0	5 (1.66%)
Fatigue	10	28	37	0	75 (24.51%)
Headache	17	31	16	0	64 (20.92%)
Hypotension	0	2	0	0	2 (0.66%)
Increased seizures	0	1	0	0	1 (0.33%)
Insomnia	5	22	18	0	45 (14.71%)
Lethargy	12	53	0	0	65 (21.24%)
Muscular weakness	4	13	12	0	29 (9.48%)
Nausea	25	11	1	0	37 (12.09%)
Pharyngitis	0	15	1	0	16 (5.23%)
Pruritus	1	0	0	0	1 (0.33%)
Pyrexia	5	1	0	0	6 (1.96%)
Rash	4	2	1	0	7 (2.29%)
Respiratory infection	0	2	0	0	2 (0.65%)
Somnolence	0	47	12	0	59 (19.28%)
Spasticity	2	3	2	0	7 (2.29%)
Tremor	15	5	0	0	20 (6.54%)
Urinary tract infection	0	3	0	0	3 (0.98%)
Urticaria	0	1	0	0	1 (0.33%)
Vertigo	21	15	5	0	41 (13.40%)
Vomiting	5	2	0	0	7 (2.29%)
Total (%)	401 (131.05%)	436 (142.48%)	142 (46.41%)	0 (0.00%)	979

## 4 DISCUSSION

The findings of this case series of fibromyalgia patients from the UKMCR show a potential association between initiation of CBMP treatment and improvement in clinical outcomes across a variety of health outcomes up until 12 months. Statistically significant improvements can be observed in validated fibromyalgia‐specific, pain, sleep, anxiety, and health‐related quality of life metrics. Furthermore, a statistically significant reduction in opioid consumption was seen at the end of follow‐up. Seventy‐two (23.53%) patients reported 979 adverse events, with the majority being either mild or moderate, and none of which were life‐threatening or disabling. However, the incidence of adverse events is higher than that typically reported across all indications for enrolment in the UKMCR.

Fibromyalgia Symptom Severity scores displayed a reduction compared to baseline up until 6 months. While this is the first study to consider this measure in relation to CBMP use, studies using the Revised Fibromyalgia Impact Questionnaire showed similar findings (Giorgi et al., 2020; Habib & Artul, 2018). Specifically, in the 2018 analysis of registry data, all 26 included patients reported a significant improvement in every questionnaire parameter (Habib & Artul, 2018). While this study has a small sample size, the unanimous effect recorded is worth noting. In a 2020 study of a similar size to the present analysis, 81.1% of patients reported moderate to significant improvement in their condition after 6 months of cannabis treatment (Sagy et al., 2019). The overall improvement in condition severity appears to be consistent across multiple observational studies, which, although promising, requires further assessment in randomized controlled trials.

In measuring overall health‐related quality of life, the EQ‐5D‐5L index values showed improvement at every follow‐up compared to baseline. Similar results were found in a study documenting an average improvement of 0.025 in the index value across a 6‐week comparison in chronic pain patients using medical cannabis (Peterson et al., 2021). In a previous analysis of the UKMCR observing chronic pain patients, including fibromyalgia, statistically significant improvement in the index value compared to baseline was seen after 1 and 3 months of CBMP treatment (Kawka et al., 2021). The present study showed similar improvements in fibromyalgia, which was maintained up to 12 months. When considering the subscales of the measure, all five domains of the EQ‐5D‐5L showed statistically significant improvement in 1‐, 3‐, and 6‐month follow‐ups. Improvement in Self‐Care is contrary to prior studies involving chronic pain cohorts that showed either no statistically significant difference or a decline in this domain (Kawka et al., 2021; Peterson et al., 2021). This further supports the proposal of a supplementary benefit specifically to the fibromyalgia cohort within chronic pain, and improvement in the central sensitivity symptoms. Further dedicated analysis according to the impact of these symptoms may help further elucidate the mechanism of action via which fibromyalgia patients derive clinical benefit after initiating CBMP treatment.

There was also a decrease in reported pain, as shown by the EQ‐5D‐5L‐Pain and Discomfort scores and VAS‐Pain scores, where there were statistically significant improvements in the pain severity at 1‐, 3‐, and 6‐month follow‐ups. In a placebo‐controlled trial involving fibromyalgia patients using THC‐rich cannabis oil lasting 8 weeks, the intervention group showed significant improvement in their pain scores (Khasabova et al., 2011), supporting the hypothesis that CBMP treatments can improve fibromyalgia‐related pain in the short term. Nabilone, a synthetic THC analogue, has also previously demonstrated analgesic effects in fibromyalgia patients (Slivicki et al., 2021). A 2006 study showed significant alleviation in both daily perceived pain and experimentally evoked pain after orally administered THC, suggesting a central mechanism of action (Schley et al., 2006). These findings support the mechanism of action proposed by preclinical studies that show CB1 agonists to promote analgesia (Khasabova et al., 2011; Slivicki et al., 2021).

Sleep quality was also improved as shown by the increase in SQS scores up to 6 months. The positive effects of CBMPs on sleep quality in the setting of fibromyalgia have been noted in various studies (Chaves et al., 2020; Fiz et al., 2011; Giorgi et al., 2020), and is a promising finding as sleep disturbance contributing to fatigue affects 76% of patients with the condition (Choy, 2015). The role of the endocannabinoid system in sleep regulation is complex with contrasting effects described in the literature (Babson et al., 2017). When compared in a randomized controlled trial to amitriptyline, a first‐line therapy in fibromyalgia, nabilone, a THC analogue, was found to be superior in improving sleep (Ware et al., 2010). However, a 2017 review of cannabis and sleep quality suggested that high‐dose CBD contributes to a decrease in sleep latency, decreases frequency of arousals during the night, and has an overall sedating effect, where low‐dose CBD is associated with increased wakefulness (Babson et al., 2017). As a result, future analyses should evaluate the effects of the THC:CBD ratio in participants to provide insight into the dose–response relationship of CBMPs and sleep quality.

There was an improvement in GAD‐7 in all follow‐ups and EQ‐5D‐5L‐Anxiety and Depression at 1 and 3 months. At least 75% of patients had either “mild” or subclinical anxiety at every follow‐up. These findings are supported by fibromyalgia patients in a controlled trial responding with significant improvement in questionnaire items “feel good” and “depression” after cannabis therapy (Chaves et al., 2020). On the other hand, in a meta‐analysis of CBMP treatment for anxiety and depression, mixed results were seen (Kosiba et al., 2019), likely to be related to the inverted U‐shaped dose–response curve observed in both animal and human studies investigating the anxiolytic effects of CBD (Guimarães et al., 1990; Linares et al., 2019; Nazario et al., 2015; Zuardi et al., 2017). These effects are supported by preclinical research that highlights the anxiolytic properties of cannabinoids mediated via effects of CB1, serotoninergic, and TRPV1 receptors of the central nervous system (Zuardi et al., 2017). The potential for CBMPs to address multiple domains of burden in fibromyalgia is a promising area of future evaluation.

The findings of this study propose an association between CBMPs and a reduction in opioid consumption. Statistically significant reduction in median oral morphine equivalent at 3 months and at extraction dates compared to baseline was observed. The high proportion of current cannabis consumers at baseline may indicate that some participants had already reduced or substituted prescribed opiates for cannabis and therefore the effects may not have been fully captured in the present analysis. The reduction is unable to imply clinical significance, as this study has not employed the Medication Quantification Scale to formally measure the negative impact of opioids on the patient cohort (Goudman et al., 2020). In a large prospective study of Canadian patients prescribed medical cannabis, a 78% reduction in mean opioid dosage was observed at the 6‐month follow‐up (Lucas et al., 2021). A systematic review similarly reported a 64%–75% reduction in opioid dose when CBMPs were used as an adjunct, but all studies had a high risk of bias (Okusanya et al., 2020). Furthermore, opioids are commonly prescribed in fibromyalgia patients, and CBMPs lower the incidence of serious adverse events and dependence in comparison (Sagy et al., 2019).

There was an incidence of 3.20 adverse events per individual in this study, where the majority were rated moderate or mild, and none were life‐threatening/disabling. A total of 35.34% of the participants in the cannabis naïve at baseline treatment group reported adverse events. Short‐term use of CBMPs has been associated with higher risks of nonserious adverse events (Wang et al., 2008), and a previous study found high‐dose THC to be associated with dissipated incidence of somnolence as treatment period increased (Gorelick et al., 2013). Furthermore, the adverse effects of THC, including anxiety, have been seen to be reversed by other cannabinoids, notably CBD (Andre et al., 2016; Russo & Guy, 2006). This points to a likelihood that these patients may see these events resolve as they continue treatment, after an initial adjustment period or alterations of their preparations.

The incidence rate of adverse events observed in this cohort was relatively high compared to analyses of other conditions using data from the UKMCR. This may be due to central sensitivity syndromes such as fibromyalgia being associated with a higher incidence of adverse events in response to medication (Liu et al., 2016). Women are also seen to report more adverse events (Watson et al., 2019), and fibromyalgia disproportionately affects women at a 2:1 ratio (Ablin & Sarzi‐Puttini, 2020; Cameron & Hemingway, 2020). Fibromyalgia is highly associated with irritable bowel syndrome, another central sensitivity syndrome, hypothesized to be the result of an altered microbiota, supported by pain reduction following treatment with antibiotics (Erdrich et al., 2020; Marum et al., 2017; Pimentel et al., 2010)—irritable bowel syndrome is also associated with an elevated drug intolerance, adverse effect incidence, and resultant treatment discontinuation (Lembo, 2004; Poitras et al., 2008). The postulation of a predisposition toward adverse events is also shown to be secondary to the nocebo effect, which accounts for 81.7% of event rate in fibromyalgia patients experiencing adverse events in active medications (Häuser et al., 2012). A proposed study to examine the effects of central sensitization in fibromyalgia in respect to treatment response may produce pertinent findings to further this discussion (ClinicalTrials.gov, 2019). Selection bias due to self‐reporting may also escalate the adverse event incidence, but in a 2021 open‐label case series, 48.6% patients reported adverse events, a twofold increase compared to the proportion of patients affected by adverse effects in the present study, suggesting that overreporting has not occurred (Mazza, 2021).

Subgroup analysis suggests that those with prior exposure to cannabis were likely to experience improvements in more domains, as well as fewer adverse events, compared to cannabis‐naïve counterparts. This suggests that despite potential to develop pharmacological tolerance to the effects of compounds contained within CBMPs, there were additional benefits derived from accessing a pharmaceutical‐grade product prescribed under supervision of expert clinicians. These additional benefits may be derived from the improved consistency of product characteristics and safety provided by CBMPs (Hazekamp, 2016). However, this may also represent a selection bias within the cohort as those who had previously consumed cannabis may be self‐selecting as responders to therapeutic properties of cannabinoids. Moreover, while these individuals were counselled against the continued consumption of illicit cannabis, it is not possible to ensure that individuals did not continue to consume illicit cannabis while in receipt of a prescription.

### 4.1 Limitations

As this study is a consecutive case series, it is economically efficient in comparison to randomized controlled trials. However, due to the lack of a control group or comparator arm, causality cannot be concluded and common biases affecting observational studies such as regression to the mean and attrition bias cannot be excluded. This is exacerbated by limited access to CBMP dose throughout titration, with analysis instead limited to maximally titrated dose of cannabinoid, limiting assessment of a dose–response relationship. The study is subject to self‐reporting bias as patients may be likely to report inflated scores due to an anticipatory effect, which is increased due to the heightened placebo effect associated with cannabis and retrospective nature of PROMs (Althubaiti, 2016). Due to the subjective nature of the PROMs collection, the outcomes are susceptible to a recall bias. As patients are not blinded to treatment, this can lead to confirmation bias. Moreover, while missing and incomplete data are inherent in registry research, this is likely to exacerbate selection bias. Additionally, unlicensed CBMPs are only available on private prescription, therefore the data may not be fully representative of the U.K. fibromyalgia population. However, the clinic covers a broad geographic area, covering the whole United Kingdom and Channel Islands. Moreover, 53.27% of participants were unemployed, suggesting a mix of socioeconomic statuses.

Another important note is the varying baselines between follow‐up comparisons across the PROMs. There is a tendency toward more positive PROMs baseline scores as follow‐up time increases, for example, the median Fibromyalgia Symptom Severity score is 23.00 (19.00–28.00) at baseline and 20.00 (16.00–27.00) at 12 months. Selection bias could explain this, where patients who were current users at baseline already experienced improvements from CBMP therapy and are more likely to continue treatment for a long time. Another factor that could contribute to this is a possible inverse relationship between severity of condition and intolerable adverse effects leading to dropout. Within the parameters of this analysis, this is not possible to conclude, but further investigation is recommended to examine a relationship. Finally, there were multiple analyses that could contribute to the likelihood of a false positive finding. To account for this, a Bonferroni correction of *p*‐values was conducted, which leads to previously statistically significant findings becoming nonsignificant after correction, limiting the likelihood of Type I error.

## 5 CONCLUSION

The results of this study suggest that there is an associated improvement in fibromyalgia‐specific symptoms, in addition to sleep, anxiety, and health‐related quality of life. Those who reported prior cannabis use appeared to have a greater response to treatment effects. In addition, nonclinically significant reductions in opioid prescribing were observed. CBMPs were largely well‐tolerated; however, the adverse event incidence in fibromyalgia was higher than other conditions captured within the UKMCR. Despite these results, the notable limitations of the study design mean that causation cannot be proven in this study. Instead randomized controlled trials are still necessary to examine this effect in earnest. However, this study adds to the clinical evidence that can inform the design of such trials, as well as current clinical practice.

## AUTHOR CONTRIBUTIONS

Mikael H. Sodergren is the principal investigator for this paper and had direct clinical responsibility for patients. Claire Wang, Simon Erridge, Carl Holvey, Ross Coomber, James J. Rucker, Michael W. Platt, and Mikael H. Sodergren contributed to the study conception and design. Claire Wang, Simon Erridge, Carl Holvey, Ross Coomber, Azfer Usmani, Mohammed Sajad, Wendy Holden, and Michael W. Platt contributed to the acquisition of data. Claire Wang, Simon Erridge, James J. Rucker, Michael W. Platt, and Mikael H. Sodergren contributed to the analysis and interpretation of data. Claire Wang, Simon Erridge, and Mikael H. Sodergren contributed to the drafting of the manuscript. Claire Wang, Simon, Erridge, Carl Holvey, Ross Coomber, Azfer Usmani, Mohammed Sajad, Wendy Holden, James J. Rucker, Michael W. Platt, and Mikael H. Sodergren contributed to critical revision. All authors agreed to be accountable for all aspects of the work and approved the final manuscript.

## CONFLICT OF INTEREST STATEMENT

Claire Wang is a medical student at Imperial College London. Claire Wang has no shareholdings in pharmaceutical companies. Simon Erridge is a junior doctor and undertakes paid consultancy work at Sapphire Medical Clinics (London). Simon Erridge is a research fellow at Imperial College London. Simon Erridge has no shareholdings in pharmaceutical companies. Carl Holvey is chief clinical pharmacist at Sapphire Medical Clinics (London). Carl Holvey has no shareholdings in pharmaceutical companies. Ross Coomber is a consultant orthopedic surgeon at St George's Hospital, London and a director at Sapphire Medical Clinics (London). Ross Coomber has no shareholdings in pharmaceutical companies. Azfer Usmani is a pain specialist at Sapphire Medical Clinics (London) and a consultant at Dartford and Gravesham NHS Trust. Azfer Usmani has no shareholdings in pharmaceutical companies. Mohammed Sajad is a pain specialist at Sapphire Medical Clinics (London) and a consultant at Dudley Group of Hospitals NHS Trust. Mohammed Sajad has no shareholdings in pharmaceutical companies. Rahul Guru is a pain specialist at Sapphire Medical Clinics (London). Rahul Guru has no shareholdings in pharmaceutical companies. Wendy Holden is a pain specialist at Sapphire Medical Clinics (London). Wendy Holden is a specialist advisor to Arthritis Action, a U.K. charity founded to help individuals with arthritis. Wendy Holden has no shareholdings in pharmaceutical companies. James J. Rucker is a consultant psychiatrist, a director, and a shareholder at Sapphire Medical Clinics (London). James J. Rucker is an honorary consultant psychiatrist at The South London & Maudsley NHS Foundation Trust, and an NIHR Clinician Scientist Fellow at the Centre for Affective Disorders at King's College London. James J. Rucker is funded by a fellowship (CS‐2017‐17‐007) from the National Institute for Health Research (NIHR). James J. Rucker leads the Psychedelic Trials Group at King's College London. King's College London receives grant funding from COMPASS Pathways PLC to undertake phase 1 and phase 2 trials with psilocybin. COMPASS Pathways PLC has paid for James J. Rucker to attend trial‐related meetings and conferences to present the results of research using psilocybin. James J. Rucker has undertaken paid consultancy work for Beckley PsyTech and Clerkenwell Health. Payments for consultancy work are received and managed by King's College London and James J. Rucker does not benefit personally. James J. Rucker has no shareholdings in pharmaceutical companies. Michael W. Platt is a consultant in pain services at Sapphire Medical Clinics (London). Michael W. Platt has no shareholdings in pharmaceutical companies. Mikael Sodergren is a consultant hepatopancreatobiliary surgeon at Imperial College NHS Trust, London. He is a senior clinical lecturer at Imperial College London. He is the Managing Director of Sapphire Medical Clinics (London). He is the Chief Medical Officer of Curaleaf International. The views expressed are those of the author(s) and not necessarily those of the NHS, the NIHR, or the Department of Health.

### PEER REVIEW

The peer review history for this article is available at https://publons.com/publon/10.1002/brb3.3072.

## Data Availability

Restrictions exist on distribution of data. For availability, please contact the corresponding author directly.
